# Levels of Salt Reduction in Bread, Acceptability and Purchase Intention by Urban Mozambican Consumers

**DOI:** 10.3390/foods11030454

**Published:** 2022-02-03

**Authors:** Neusa Jessen, Albertino Damasceno, Patrícia Padrão, Nuno Lunet

**Affiliations:** 1 EPIUnit-Instituto de Saúde Pública, Universidade do Porto, Rua das Taipas nº 135, 4050-600 Porto, Portugal; neusa.jessen@gmail.com (N.J.); tino_7117@hotmail.com (A.D.); padraopatricia@gmail.com (P.P.); 2 Laboratório Para a Investigação Integrativa e Translacional em Saúde Populacional (ITR), Rua das Taipas, nº 135, 4050-600 Porto, Portugal; 3 Departamento de Medicina, Hospital Central de Maputo, Avenida Eduardo Mondlane 1653, Maputo 1100, Mozambique; 4 Departamento de Ciências da Saúde Pública e Forenses e Educação Médica, Faculdade de Medicina da Universidade do Porto, Alameda Prof. Hernâni Monteiro, 4200-319 Porto, Portugal; 5 Faculdade de Ciências da Nutrição e Alimentação, Universidade do Porto, Rua Dr. Roberto Frias, 4200-465 Porto, Portugal

**Keywords:** sodium, salt, bread, perception, acceptability, purchase intention, sensory evaluation, Mozambique

## Abstract

Excess sodium (Na) consumption is implicated in several health problems, particularly hypertension, and bread is an important dietary source. We aimed to analyze perception of salt, acceptability, and purchase intention of low-salt and unsalted white bread by consumers in Mozambique. Sensory evaluation was performed using a triangular test (N = 42) to perceive if differences in saltiness were detected when comparing low-salt and unsalted with salt-reduced white bread. Nine-point hedonic and five-point purchase intention scales were used to measure acceptability and purchase intention, respectively (N = 120). Difference in saltiness was not detected when fresh white bread with 282 mg Na/100 g vs. 231 mg Na/100 g and 279 mg Na/100 g vs. 123 mg Na/100 g were compared. Difference in saltiness was not detected when comparing unsalted vs. 64 mg Na/100 g, while differences were detected when unsalted vs. 105 mg Na/100 g and unsalted vs. 277 mg Na/100 g were compared. Overall acceptability and purchase intention were not affected by reductions of Na in bread. A reduction of up to more than 50% of Na was not perceived and a small level of Na was not distinguished from unsalted bread. Consumers were shown to accept and be willing to buy both unsalted and salt-reduced bread, suggesting that Na can be reduced from current levels.

## 1. Introduction

Excessive dietary salt is an important preventable risk factor for several health problems [1,2,3,4,5,6,7]. The absolute global burden due to high Na intake increased significantly during the last three decades (from 1990 to 2019), with an estimated rise in the total number of attributable deaths and disability-adjusted life-years (DALYs) of around 0.57 million and 11.48 million, respectively [8].

High Na intake has been especially associated with cardiovascular morbidity and mortality [7,9,10,11,12,13], with a direct influence on high blood pressure and probably on stroke mortality [14,15,16,17,18,19].

The World Health Organization (WHO) recommends a maximum daily intake of 2000 mg of Na (equivalent to 5000 mg of salt) [20]. However, overall high intakes have been found in several populations [21], including in Africa, and particularly in urban settings [22,23]. In Mozambique, the mean 24-h urinary Na excretion, evaluated in a sample of Maputo dwellers, was found to be twice the recommended by the WHO [24]. Reducing salt intake at the population level has been shown to be a cost-effective and cost-saving intervention [25,26,27], and reformulation of processed foods is one of the three core pillars recommended by the WHO to promote such reduction [28]. Bread is a common target of food reformulation. This is not surprising given that it is frequently rich in salt and accessible, representing an important part of the daily diet of several populations [29], including the Mozambican [24,30].

In fact, in Mozambique, a study performed in 2012 evaluated the salt content of white bread available for purchase in the city of Maputo and found a mean Na level of 450.3 mg Na/100 g of white bread [31]. However, a high variability in salt content in white bread was observed between bakeries, which is in line with the lack of legislation or a formal recommendation for the amount of salt that is added during bread preparation. In a new evaluation performed in 2018, the mean Na content in white bread was lower by just over 10%, and other varieties of bread were also available, including unsalted bread [32], showing that some consumers actually choose salt reduced bread.

Food choice is highly influenced by taste. Perception of salt taste depends on the activation of taste receptors and the subsequent production of electrical impulses that are sent to the brain. This process requires a certain concentration of Na (the recognition threshold) below which the electrical signal may not be enough to elicit saltiness. When Na is reduced from food, several flavor effects are affected and a wide range of complex taste interactions occur [33]. The stealth approach, consisting of a gradual reduction of salt in foods, modifies the salt taste experience of consumers over time until it goes unnoticeable. This strategy has shown promising results in the UK population, has the advantage of not depending on behavior change, and can allow for large Na reductions in foods [33]. In fact, previous studies have shown that taste adapts to lower salt concentrations in food [34] and, in experiences with salt reduced bread, consumers were not able to detect differences in saltiness, maintaining acceptability [35,36]. However, such evaluations are not available in Mozambique and setting-specific data are needed, given the recognized differences in dietary habits and taste preferences across populations. As such, we aimed to assess the levels of salt reduction in bread that urban Mozambican consumers could detect, as well as acceptability and purchase intention of bread with different salt contents.

## 2. Material and Methods

### 2.1. Bread Production

In July of 2018, a researcher observed the usual process of bread production in a local bakery and registered all ingredients, the weights, and the steps that were followed. Subsequently, taking into account that 1 g of salt contains 400 mg of Na, five batches of white bread were produced using the same recipe but changing the amount of salt added, in order to produce breads with different concentrations of Na (according to estimates for what was to be tested). The recipe consisted of: 1 kg of white bread flour (locally refined wheat flour, from the ^®^MEREC industries SA [37]), 0.1% of additive (1 g, ascorbic acid), 3% of bread yeast (30 g), 70% water (700 g) and the desired amount of salt. All ingredients were carefully weighed using a precision automatic scale, the salt was dissolved in the water, and the dough was prepared by adding and mixing the ingredients with an automatic spiral dough mixer machine, as follows: first, the flour, the salted water, and the additive were mixed together for 30 min, then the yeast was added and mixed for an additional 10 min. The dough was divided in eight equal parts and left to rise for 45 min before baking. Once ready, each loaf of bread weighed around 220 g. The bread loaves were let to cool down and, from each batch of eight breads, four were homogenized with crust and four without crust. A sample of each homogenized bread was collected, frozen and sent for Na analysis at the University of Porto, in Portugal. Results from such analysis were used for the production of breads for the testing sessions.

### 2.2. Preparation and Analysis of Bread for the Testing Sessions

Bread for the tests was produced in the afternoon of the day before the testing session, in the same local bakery, following the same procedures of bread production. The amount of salt added to each type of bread produced for the testing sessions was based on the Na content in bread that was found in the laboratory analysis. Each batch was composed of 12 breads (with around 150 g each) with a specific salt concentration. Once baked, the bread was let to cool down, six breads were homogenized (three with crusts and three without crusts) and samples of 30 g were taken from each, packed, frozen, and sent for analysis at the University of Porto. The remaining six breads were cut and stored in labelled plastic boxes until the testing session.

The homogenized bread samples were defrosted at room temperature and two aliquots, of approximately 2 g, were collected from each sample of bread and analyzed for Na content, using flame photometry (flame photometer model PFP7; JenWay^®^, Dunmow, UK) according to a previously validated method [38]. Two readings were taken from each of the two aliquots and the mean of the results from the three samples (of the same type of bread, without crust) was used for data analysis. The Na concentrations that are presented throughout the manuscript correspond to the mean results obtained from the laboratory analysis of the samples without crust.

### 2.3. Selection of Participants

Participants were dwellers in the city of Maputo, the capital and most urbanized city of Mozambique, recruited among adult employees of the Central Hospital of Maputo. Convenience samples were selected, aiming for similar proportions of males and females, in the age-groups 18–44 years and ≥45 years, and with a number of complete years of education, <9 and ≥9 years, as described in Table 1. Pregnant women were not included. Most individuals participated in the evaluations conducted in more than one day.

Habits of bread consumption were also assessed in the beginning of the test. The proportion of participants who reported eating bread every day ranged from 85.7% to 90.8% across all sessions and the number of breads eaten per day ranged from half (around 75 g) to four breads (around 600 g), with just above 50% of participants consuming at least one bread per day (around 150 g).

The present study was conducted in accordance with the Declaration of Helsinki Ethical Principles. Ethical approval was granted by the Ethics Committee of the Faculty of Medicine of University Eduardo Mondlane in Maputo (CIBIS-FM & HCM/074/2018) and written informed consent was obtained from all participants. The scientific direction of the Central Hospital of Maputo consented to the participation of employees and for the study to be conducted within the Hospital premises.

### 2.4. Bread Testing Sessions

This study comprised sensory evaluation [39] through difference testing, to assess differences in the levels of salt in bread that consumers could detect, and acceptability and purchase intention testing of bread with different salt contents. The former focused on the comparison of samples of fresh bread with Na levels at around 280 mg/100 g with samples with gradually lower concentrations (≈230 mg/100 g and ≈120 mg/100 g), as well as on the comparison of samples of fresh bread with no added salt with samples with increasing Na concentrations (≈60 mg/100 g, ≈100 mg/100 g, ≈280 mg/100 g). The latter focused on the evaluation of samples with different Na levels, namely ≈ 290 mg/100 g, 215 mg/100 g, ≈170 mg/100 g, ≈80 mg/100 g, ≈60 mg/100 g, and no added salt (Table 1).

For the testing sessions, samples were prepared by slicing the fresh loaf of bread, discarding the ends, cutting each slice in half and removing the crusts. Multiple three-digit codes were randomly selected and assigned to each sample by labelling individual white plates (Supplementary Figure S1). Therefore, different samples from the same bread received different codes and, as such, participants seated next to each other received samples with different codes.

Each participant received three samples of bread per session, that were presented simultaneously [40]. To prevent presentation bias, the order of presentation of the samples was randomized and balanced across participants.

Participants were given general information regarding the objectives of the study and instructed to taste the samples starting from the left, to drink some water between the samples to cleanse the palate, and then to mark their answer in the paper form that was provided.

#### 2.4.1. Difference Testing

Difference testing was accomplished through the triangular test methodology [39,40], between September and October 2019. A total of 42 participants were involved in each of the five days of testing sessions as described in Table 1. The testing sessions took place once a week, during the morning (six sessions with seven persons in each, in a room of the Central Hospital of Maputo, organized with individual tables and chairs (Supplementary Figure S1).

Participants received three samples, two from the same bread and another from a bread with a different salt content; each sample was identified with a random three-digit code. Using the letters A and B to represent the order of presentation of the least and the more salted samples, respectively, the possibilities of presentation were: ABB, BAB, BBA, AAB, ABA, BAA. Participants were told that one of the samples that they were about to taste was different, either had more or less salt, were instructed to taste the three samples and then select the odd sample among them using the form provided (Supplementary Figure S1). This is a forced choice test and participants had to give an answer even if they were not sure of the difference.

#### 2.4.2. Acceptability and Purchase Intention Testing

Acceptability and purchase intention testing were performed after the conclusion of difference testing, in December of 2019. A total of 120 participants were involved in each of the two days of testing sessions (six sessions with 10 participants in the morning and six sessions in the afternoon), which took place in the same week, two days apart (Table 1).

Assessors tasted three samples of bread with different salt concentrations; each sample was identified with a random three-digit code. Using the letters A, B and C to represent samples with different concentrations of Na, the possibilities of presentation were: ABC, CAB, BCA, BAC, ACB, CBA. They were told that they were about to taste breads with different salt contents and instructed to taste the one at a time and to rate the first sample regarding acceptability and purchase intention by filling the forms provided.

Acceptability was evaluated using a Portuguese version of the nine-point hedonic scale [41], where: 9 = ‘like extremely’, 8 = ‘like very much’, 7 = ‘like moderately’, 6 = ‘like slightly’, 5 = ‘neither like nor dislike’, 4 = ‘dislike slightly’, 3 = ‘dislike moderately’, 2 = ‘dislike very much’; and 1 = ‘dislike extremely’.

Purchase intention was evaluated using a Portuguese version of a five-point scale, where: 5 = ‘definitely buy’, 4 = ‘probably buy’, 3 = ‘might or might not buy’ 2 = ‘probably not buy’; and 1 = ‘definitely not buy’.

### 2.5. Statistical Analysis

For the difference test, a sample size of 42 was calculated, to be able to detect differences of at least 25% in Na content in relation to the probability of detecting the odd sample by chance (one third), for a confidence level of 95% and a power of 90%. Binomial probability distribution was used, with a probability of success of one third, to calculate the upper critical value or the maximum number of correct identifications in the triangular test until statistical significance; this value was found to be 19. The Chi-squared test was used to compare the difference in the number of correct identifications according to demographic characteristics.

For the acceptability test, to estimate a difference between sample means of 0.8 on the nine-point hedonic scale (10% of the sensory scale), assuming a confidence level of 95%, a power of 90%, and a root mean square error divided by the scale length (RMSL) of 0.23 (averaged from other studies) with a standard deviation of 0.037, a sample of 112 consumers was needed [42]. We assembled a sample of 120 participants.

Mean scores were calculated for acceptability and purchase intention of each sample, overall and according to socio-demographic variables. For acceptability and purchase intention testing, one-way ANOVA was used when variances were homogeneous, or the Welch test otherwise, to evaluate if there was a significant difference in mean scores across the bread types. Independent samples *t*-test was used to determine if the mean scores of acceptability and purchase intention were significantly different according to gender, age, or level of education.

Analyses were conducted using IBM^®^ SPPSS^®^ statistics package, version 26, and, for all analyses, statistical significance was set at the 0.05 level.

## 3. Results

### 3.1. Difference Testing

**Figure 1:**
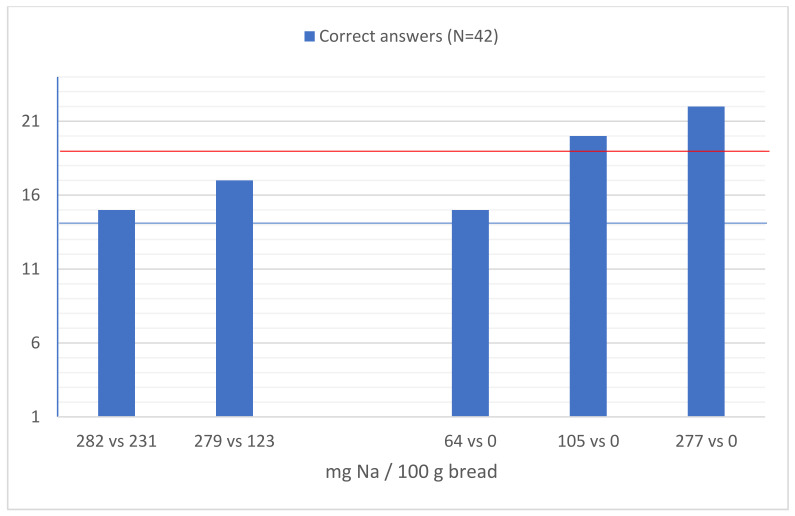
Results from difference testing; 14-number of correct answers by chance; 19-maximum number of correct answers until statistical significance.

Results from difference testing are depicted in Figure 1. The odd sample was correctly identified by 15/42 participants (35.7%) when 282 mg Na/100 g vs. 231 mg Na/100 g were compared, and by 17/42 participants (40.5%) when comparing 279 mg Na/100 g vs. 123 mg Na/100 g. In both cases the proportion of correct answers was not significantly different from the 33.3% that are expected by chance in the triangular test. When comparing fresh bread samples with no added salt with samples with 64 mg Na/100 g, participants did not detect the difference between samples (15/42 correct answers, 35.7%). The odd sample was correctly identified by 20/42 participants (47.6%) when samples with no added salt vs. 105 mg Na/100 g were compared, and by 22/42 participants (52.4%) when comparing samples with no added salt vs. 277 mg Na/100 g. No consistent statistically significant differences were observed between males and females, according to age (< or ≥45 years old) or level of education (< or ≥ 9 years) (Table 2).

### 3.2. Acceptability and Purchase Intention Testing

**Figure 2:**
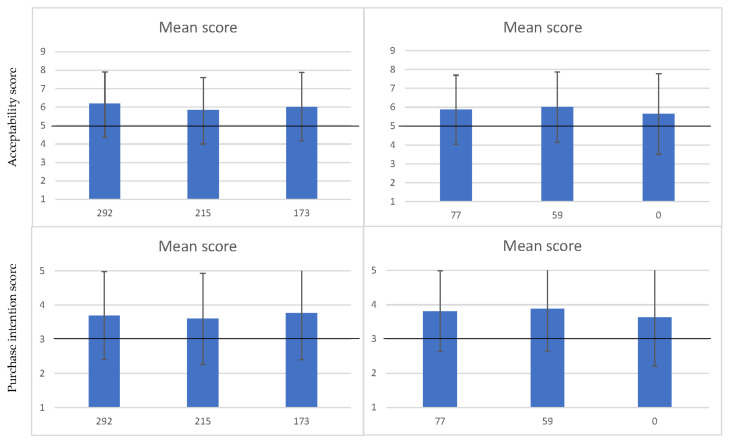
Mean scores for acceptability and purchase intention testing.

Results from acceptability and purchase intention testing are depicted in Figure 2. When comparing fresh bread samples with 292 mg Na/100 g, 215 mg Na/100 g and 173 mg Na/100 g, the mean acceptability scores (6.18, 5.83, and 6.00, respectively; *p* = 0.334), and the mean purchase intention scores (3.69, 3.60, and 3.77, respectively; *p* = 0.612) were not significantly different. When comparing fresh bread samples with no added salt, 59 mg Na/100 g and 77 mg Na/100 g, the mean acceptability scores (5.64, 6.01, and 5.87, respectively; *p* = 0.339), and the mean purchase intention scores (3.63, 3.88, and 3.81, respectively; *p* = 0.312) were not significantly different. No consistent statistically significant differences were observed between males and females, according to age (< or ≥45 years old) or level of education (< or ≥ 9 years) (Table 2).

## 4. Discussion

In the present study, participants were not able to detect the difference between samples with ≈280 mg Na/100 g fresh white bread compared with samples with less 18% and 56% of Na. Moreover, participants could not detect the difference in saltiness when unsalted fresh white bread was compared with samples with 64 mg Na/100 g fresh white bread. On the other hand, when comparing unsalted fresh white bread with 105 mg Na/100 g and with 277 mg Na/100 g, participants detected the difference, suggesting that in the lower extreme of Na in bread (increasing from unsalted), consumers detect saltiness more easily than in the upper extreme, where greater reductions could be made without being noticed. However, the acceptability and purchase intention did not decrease by reduction of salt or even by completely removing salt from bread.

Studies measuring consumer’s acceptability of salt-reduced bread are scarce and most of the available studies were conducted in high-income countries [43]. Even so, in line with our findings, a previous study conducted in an indigenous Australian community, comparing fresh white bread with 400 mg Na/100 g with reductions of 12.5% and 25% of Na, found that consumers did not detect differences and liking of the bread did not change [36]. Furthermore, in Australia, in a randomized controlled study, cumulative reductions of 5% in salt content in bread were performed weekly for six consecutive weeks and acceptability was still similar to the control group [35]. Additionally, a gradual weekly reduction of up to 52% of Na, from 498 mg Na/100 g bread, was evaluated, showing that consumption of bread did not decrease and consumers did not compensate the lack of Na with saltier sandwich fillings [44].

The choice of food may be influenced by several factors, including individual taste perception. The possible influence of intrinsic (including genetics, gender, age, and ethnicity) and extrinsic factors (such as health conditions, medications, smoking, and weight) in the function of taste is not comprehensively described in the literature and while some studies found associations, others have not [45]. In their study, Puputti et al. [45] reported a reduced taste sensitivity among males (compared to females) and among older individuals. In a systematic review of studies on taste perception among young and older adults, the authors found that, although some studies corroborate the hypotheses of a decline in the sense of taste with ageing, the extent of such a decline was variable [46]. Perception of the intensity of saltiness has been related to socio-demographic characteristics, with some studies suggesting that elderly age and male gender are associated with reduced sensitivity to saltiness [45,47]. However, in the present study, the number of participants in each testing session was balanced with respect to gender and age groups and, in line with results from the study by McMahon et al. [36], we did not find consistent differences on saltiness perception and acceptability of the salt reduced bread according to sex and age. Additionally, higher serving temperature of the food seems to reduce sensitivity to saltiness among consumers used to eating food at lower temperatures [48], but, in our study, bread was served at room temperature in all sessions. The habit of smoking and male gender have also been associated to increased preference for salty foods [49], although others did not found association of smoking status with taste recognition or sensitivity [45]. Nevertheless, in the present study, among the participants of the acceptability test, the proportion of current smokers was less than 7% (5/120 in the first day and 8/120 in the second day). Moreover, there were no statistically significant differences in perception of saltiness, acceptability, and purchase intention, according to level of education.

A modest reduction of salt intake, made over four or more weeks, was shown to significantly reduce blood pressure in both hypertensives and normotensives [19] and may reduce the risk of stroke and coronary heart disease in adults [9]. This is especially important in Mozambique, a low resource setting, with a very high prevalence of hypertension (39% in 2014/2015) [50] but where a high proportion of hypertensives not on pharmacologic treatment were classified at low (<10%) 10-year cardiovascular (CV) risk [51]. Taking into account that the CV risk associated with high blood pressure begins at values as low as 115 mmHg of systolic blood pressure and only half of hypertensive adults in Mozambique were found to be eligible for pharmacological treatment (according to WHO guidelines) [51], it would be important to start implementing potentially effective and cost-saving lifestyle interventions to prevent and control hypertension, such as salt intake reduction [7,25,26]. These should be urgently implemented in urban settings of the country, since hypertensives from urban areas have been shown to present higher total CV risk [51], and they should be progressively extended to rural areas. Several countries around the world are successfully implementing food reformulation to decrease salt content and reduce salt intake at the population level, and bread is the most commonly, and usually the first, targeted food product [52]. In fact, voluntary or mandatory targets for maximum Na content in bread have been defined in several countries [53,54,55,56,57], including in South Africa, where a regulation was set for a target of <380 mg Na/100 g [57]. Although such recommendations are not yet available in Mozambique, in a recent evaluation of trends in salt content of white bread produced in Maputo city from 2012 to 2018, we found that voluntary reduction is possible and actually occurred [32]. Nevertheless, with the current mean level of Na in white bread produced in Maputo of 419 mg/100 g [32], bread may be contributing with just over 20% for the maximum daily recommendation of Na intake for adults and even more for children [20]. The present study shows that such levels of Na can be further reduced. Additionally, establishing a target level would be important to guide producers and to reduce the large variability of the current Na levels observed across breads available in different bakeries. Voluntary reformulation may be the fastest and easiest starting point, but concerns regarding reduction of sales may arise among manufacturers and lead to resistance to change, especially due to the effects of Na reduction on palatability and on shelf life [58]. Nonetheless, it has been shown that although the complete removal of salt from bread leads to alterations on crumb structure starting on the fifth day after production, the quality of the bread is conserved even when very small levels of salt are added [58,59]. In fact, in the present study, the final bread quality was kept when salt was reduced or even completely removed, so acceptability and purchase intention of local consumers did not change. Even so, gradual reductions of Na in bread over time may be a better strategy to allow time for adaptation of taste sensors of the consumers. Furthermore, corroborating our results is the fact that unsalted bread is currently available for purchase in some bakeries of the city [32], attesting the technical feasibility and suggesting that other manufacturers may be convinced to widen their offer to include this variety of bread.

To the best of our knowledge, the present study is the first to assess perception of saltiness in bread, acceptability, and purchase intention of salt-reduced bread by Mozambicans, an African population [43,60]. Sound methodologies for sensorial analysis were selected, and a standardized experimental protocol was carefully elaborated and consistently followed during all the study, according to the principles of good practice that are recommended for each of the selected tests. As recommended for acceptability tests [40], a panel of bread consumers with no sensory training was selected and the local language was used for the test sessions. Even so, some limitations must be discussed. One of which is the fact that only adult consumers were included and, as such, the extrapolation of results to adolescents and children may not be possible. Moreover, the study was conducted with a sample of urban Maputo inhabitants and results may not directly apply to rural regions of the country or to other countries, where nutrition habits may vary. Additionally, since Na concentrations differ between crusts and the inner part of bread, to increase the homogeneity, only the inner part of the bread was used for testing and, as a consequence, the maximum tested concentration was 292 mg of Na/100 g. Thus, it is not known if perception would be the same if consumers ate the bread with crusts. Nevertheless, in this kind of bread the crust is slim, and we do not expect that tasting the bread with crust would have a large impact on results. In addition, it would be interesting to evaluate the averages of the scales by attributes of each bread, but since we were using untrained consumers as assessors, we did not rate each attribute of the bread (such as appearance, color, texture and softness), just the overall liking.

The present study adds to previous research on this topic by providing information on perception of saltiness and acceptability of low-salt bread from a sample of a southern African population, from a country located in the east coast, with a tropical climate. Most people live in the coastline and there are several salt producers, including big, registered industries and small, unregistered producers. Salt is widely available and used for seasoning (during cooking and on already cooked food) and to preserve food. Structural and population-level interventions present greater potential for salt intake reduction by this population [61] and the present study contributes with important guiding information. Nevertheless, since reduction of salt in bread may change its physical characteristics and disrupt the sensory profile, it would be important to obtain reliable quantitative information, based in instrumental analysis, to reassure manufacturers about the safety and quality of the salt-reduced bread. As such, further research is needed to better understand the effects of salt reduction in perishability of bread produced locally, to analyze if local consumers would adopt saltier fillings to compensate the reduction of Na in bread, and to evaluate perception and acceptability of salt reduced bread by children and adolescents and among rural dwellers. As future perspectives, after the evidence raised by the present study, the capacity to perform instrumental analysis and to address technological properties of salt-reduced bread in local laboratories should be pursued. Apart from improving our understanding of the influences that reducing salt in bread have on the technological processes of breadmaking and on the final attributes of the bread produced, applying instrumental analysis may provide important information for bread producers and for future comparisons with findings from different populations. 

## 5. Conclusions

This study shows that it is possible to reduce the current level of Na in bread produced in Mozambique, keeping acceptability and purchase intention. Local consumers were not able to detect reductions of up to more than 50% of Na concentration and acceptability and purchase intention did not change when salt was reduced or completely removed from bread. Even when consumers detected the difference in salt content between breads, acceptability and willingness to purchase did not change significantly. Findings from the present research can be used by programs of nutrition and prevention and control of Non-Communicable Diseases of the Ministry of Health of Mozambique to define recommendations and influence the production of low-salt bread, to define bread procurement standards for public institutions, and to improve the food environment in the urban centers of the country.

## Figures and Tables

**Table 1:** Tests performed and sociodemographic characteristics of participants.

Test Applied	Difference Testing *					Acceptability Testing	
Experiment Performed	Salt Reduction		Salt Increase			Different Salt Leves (High)	Different Salt Leves (Low)
Day of experiment	# 1	# 2	# 3	# 4	# 5	# 6	# 7
Na concetrations tested (mg Na/100 g bread)	282 → 231	279 → 123	0 → 64	0 → 105	0 → 277	292 vs. 215 vs. 173	77 vs. 59 vs. 0
Participants (N)	42	42	42	42	42	120	120
Female (%)	50%	50%	52.4%	50%	50%	55.8%	53.3%
Age, mean years (SD)	46 (11.2)	45 (12.3)	46 (11.3)	45 (12.1)	46 (11.4)	41 (10.6)	43 (11.0)
Level of education < 9 ^†^ (%)	47.6%	50%	50%	50%	50%	38.3%	48.3%

SD, standard deviation; * Triangular test; ^†^ Years of school completed.

**Table 2:** Comparison of correct answers in difference testing and scores of acceptability and purchase intention testing, according to demographic variables.

	Gender			Age (Years Old)			Level of Education *		
Test	Female	Male	*p*	18–44	≥45	*p*	<9	≥9	*p*
Difference ^†^ (n/N) ^‡^									
282 vs. 231	9/21	6/21	0.334	8/21	7/21	0.747	5/20	10/22	0.167
279 vs. 123	8/21	9/21	0.753	9/21	8/21	0.753	11/21	6/21	0.116
105 vs. 0	12/21	8/21	0.217	14/21	6/21	0.013	7/21	13/21	0.064
64 vs. 0	10/22	5/20	0.167	6/21	9/21	0.334	8/21	7/21	0.747
277 vs. 0	10/21	12/21	0.537	10/21	12/21	0.537	11/21	11/21	1.000
Acceptability [Mean score (SD)]									
292	6.52 (1.75)	6.21 (2.15)	0.615	6.38 (1.94)	6.36 (1.98)	0.966	6.57 (1.87)	6.27 (1.99)	0.643
215	6.16 (1.70)	6.00 (1.77)	0.778	6.29 (1.51)	5.67 (2.10)	0.299	5.63 (1.93)	6.42 (1.50)	0.153
173	5.71 (1.87)	6.47 (1.68)	0.187	5.96 (1.91)	6.29 (1.64)	0.594	6.56 (1.46)	5.57 (1.96)	0.166
77	6.00 (1.61)	5.47 (2.29)	0.403	5.60 (2.09)	5.90 (1.86)	0.634	5.65 (2.01)	5.85 (1.95)	0.751
59	6.27 (1.48)	5.00 (2.09)	0.030	6.08 (1.44)	5.07 (2.34)	0.098	5.53 (2.06)	5.86 (1.71)	0.583
0	5.71 (2.28)	5.79 (2.39)	0.919	5.75 (2.36)	5.75 (2.29)	1.000	5.84 (2.41)	5.67 (2.27)	0.814
Purchase intention [Mean score (SD)]									
292	4.09 (0.89)	3.89 (1.15)	0.539	4.19 (0.85)	3.64 (1.22)	0.102	3.71 (1.20)	4.15 (0.88)	0.194
215	3.50 (1.41)	4.07 (1.16)	0.202	3.64 (1.34)	3.91 (1.38)	0.583	3.87 (1.25)	3.63 (1.41)	0.589
173	3.38 (1.49)	3.95 (1.31)	0.213	3.88 (1.48)	3.21 (1.25)	0.158	3.62 (1.15)	3.67 (1.61)	0.929
77	3.95 (1.07)	3.26 (1.09)	0.052	3.70 (0.98)	3.55 (1.28)	0.679	3.55 (1.23)	3.70 (1.03)	0.679
59	4.05 (1.21)	3.50 (1.20)	0.164	3.92 (1.19)	3.60 (1.30)	0.430	3.68 (1.42)	3.90 (1.04)	0.576
0	3.81 (1.37)	3.68 (1.42)	0.777	3.71 (1.46)	3.81 (1.28)	0.818	3.58 (1.39)	3.90 (1.38)	0.461

SD, standard deviation; * Years of school completed; ^†^ Triangular testing (*n* = 19 is the maximum number of correct identifications until statistical significance); ^‡^ Number of correct answers/total.

## Data Availability

Data is contained within the article.

## Supplementary Materials

The following supporting information can be downloaded at: https://www.mdpi.com/article/10.3390/foods11030454/s1, Figure S1: Images of the testing sessions.
