# Supplementary material for: Levels of Salt Reduction in Bread, Acceptability and Purchase Intention by Urban Mozambican Consumers
Source: Foods. 2022 Feb 3;11(3):454. doi: 10.3390/foods11030454 (PMC8834232; doi:10.3390/foods11030454)
Supplement: Supplementary file 1 [file foods-11-00454-s001.zip › foods-1482605-supplementary.pdf]

Supplementary material

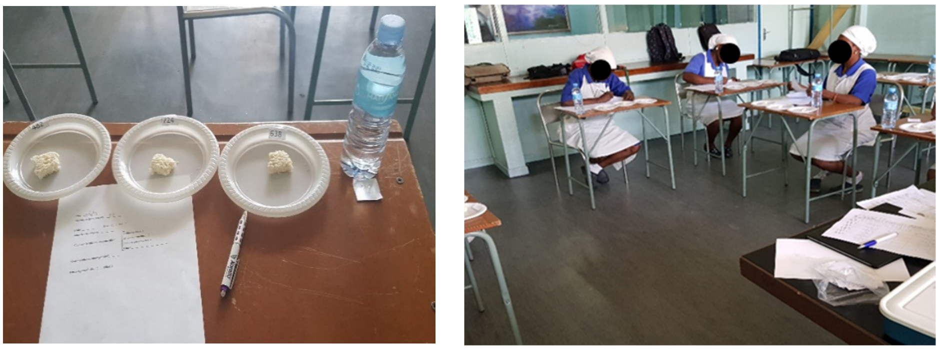

(**a**) (**b**)

Figure S1. Images of the testing sessions; a – Presentation of samples; b – Test session.
